# A Comparison between Different Methods of Estimating Anaerobic Energy Production

**DOI:** 10.3389/fphys.2018.00082

**Published:** 2018-02-08

**Authors:** Erik P. Andersson, Kerry McGawley

**Affiliations:** Swedish Winter Sports Research Centre, Department of Health Sciences, Mid Sweden University, Östersund, Sweden

**Keywords:** anaerobic capacity, cross-country skiing, endurance exercise, energetics, oxygen deficit, oxygen demand, oxygen uptake

## Abstract

**Purpose:** The present study aimed to compare four methods of estimating anaerobic energy production during supramaximal exercise.

**Methods:** Twenty-one junior cross-country skiers competing at a national and/or international level were tested on a treadmill during uphill (7°) diagonal-stride (DS) roller-skiing. After a 4-minute warm-up, a 4 × 4-min continuous submaximal protocol was performed followed by a 600-m time trial (TT). For the maximal accumulated O_2_ deficit (MAOD) method the $\dot{\text{V}}$O_2_-speed regression relationship was used to estimate the $\dot{\text{V}}$O_2_ demand during the TT, either including (4+Y, method 1) or excluding (4-Y, method 2) a fixed Y-intercept for baseline $\dot{\text{V}}$O_2_. The gross efficiency (GE) method (method 3) involved calculating metabolic rate during the TT by dividing power output by submaximal GE, which was then converted to a $\dot{\text{V}}$O_2_ demand. An alternative method based on submaximal energy cost (EC, method 4) was also used to estimate $\dot{\text{V}}$O_2_ demand during the TT.

**Results:** The GE/EC remained constant across the submaximal stages and the supramaximal TT was performed in 185 ± 24 s. The GE and EC methods produced identical $\dot{\text{V}}$O_2_ demands and O_2_ deficits. The $\dot{\text{V}}$O_2_ demand was ~3% lower for the 4+Y method compared with the 4-Y and GE/EC methods, with corresponding O_2_ deficits of 56 ± 10, 62 ± 10, and 63 ± 10 mL·kg^−1^, respectively (*P* < 0.05 for 4+Y vs. 4-Y and GE/EC). The mean differences between the estimated O_2_ deficits were −6 ± 5 mL·kg^−1^ (4+Y vs. 4-Y, *P* < 0.05), −7 ± 1 mL·kg^−1^ (4+Y vs. GE/EC, *P* < 0.05) and −1 ± 5 mL·kg^−1^ (4-Y vs. GE/EC), with respective typical errors of 5.3, 1.9, and 6.0%. The mean difference between the O_2_ deficit estimated with GE/EC based on the average of four submaximal stages compared with the last stage was 1 ± 2 mL·kg^−1^, with a typical error of 3.2%.

**Conclusions:** These findings demonstrate a disagreement in the O_2_ deficits estimated using current methods. In addition, the findings suggest that a valid estimate of the O_2_ deficit may be possible using data from only one submaximal stage in combination with the GE/EC method.

## Introduction

In short-duration endurance events, such as middle-distance running and sprint cross-country skiing, performance is not only related to the rate of aerobic energy supply but also to anaerobic energy provision (Duffield et al., 2005; Losnegard et al., 2012). This is also the case, at least to some extent, during longer-duration endurance events performed over undulating terrains and at fluctuating intensities, since the oxygen uptake ($\dot{\text{V}}$O_2_) demand will at times exceed the maximal oxygen uptake ($\dot{\text{V}}$O_2max_) (Norman et al., 1989; Skiba et al., 2012). In comparison to aerobic energy supply, anaerobic energy supply has a far more limited capacity (i.e., limited amount of energy that can be produced anaerobically) but can provide adenosine triphosphate at a much faster rate than the oxidative pathway. Therefore, the relative contribution of anaerobic energy production decreases with exercise duration (Gastin, 2001). In highly-trained runners, for example, anaerobic metabolism has been shown to account for ~34 and ~16% of the total energy turnover during 800-m and 1500-m events (lasting ~1:53 and ~3:55 min), respectively (Spencer and Gastin, 2001). In addition to energy supply, the efficacy of converting metabolic energy to external work (i.e., gross efficiency, GE) is an important component of endurance performance (Joyner and Coyle, 2008).

Energetic contributions from aerobic pathways during whole-body exercise can easily be quantified using measurements of $\dot{\text{V}}$O_2_, while a direct quantification of the anaerobic energy yield is more complicated and only possible via sophisticated, invasive and expensive technologies (Bangsbo et al., 1990). Therefore, a common approach during whole-body exercise is to indirectly estimate anaerobic energy production using the maximal accumulated oxygen (O_2_) deficit (MAOD) method (Medbø et al., 1988), which is based on calculating a linear relationship between submaximal $\dot{\text{V}}$O_2_ and speed (or power output) and estimating $\dot{\text{V}}$O_2_ demand at supramaximal speeds by extrapolation.

A main point of contention regarding the MAOD method appears to be in how best to construct the linear relationship. For example, Bickham et al. (2002) recommend 4 × 4-min stages combined with a forced Y-intercept to include baseline $\dot{\text{V}}$O_2_, while Medbø et al. (1988) proposed that 10 × 10-min stages are needed from low to high submaximal intensities, or alternatively, 2 × 10-min submaximal stages close to $\dot{\text{V}}$O_2max_ if combined with a Y-intercept. Moreover, Bangsbo (1992, 1996a,b) has criticized linear extrapolation from submaximal $\dot{\text{V}}$O_2_-speed relationships when using the MAOD method, claiming that this technique may underestimate the $\dot{\text{V}}$O_2_ demand and the accumulated O_2_ deficit during supramaximal exercise due to an exponential increase in $\dot{\text{V}}$O_2_ from low to high exercise intensities. Another problem with the MAOD method is that potential changes in substrate utilization during submaximal exercise are not considered (Fletcher et al., 2009). As such, a metabolic rate vs. speed relationship may be more appropriate when calculating MAOD, since the different energetic equivalents for fat and carbohydrate are then taken into account (Weir, 1949).

In addition to the various MAOD methods, total metabolic demand during supramaximal exercise can be estimated by multiplying the submaximal energy cost (EC) of locomotion by speed (di Prampero, 2003). In sports where external power can be determined or estimated, such as cycling and cross-country roller-skiing (Sandbakk et al., 2011; Noordhof et al., 2013; Andersson et al., 2017), submaximal GE can also be used to estimate the total metabolic demand during supramaximal exercise by dividing power output by GE (Noordhof et al., 2011; de Koning et al., 2012; Andersson et al., 2017). For both the GE and EC methods, the difference between the total accumulated metabolic demand and the accumulated aerobic energy production represents an estimate of anaerobic energy production and is usually expressed in joules, or as a VO_2_ equivalent (i.e., O_2_ deficit; Noordhof et al., 2011; Andersson et al., 2017).

In a standardized laboratory environment, where air drag is usually negligible, the GE and EC methods of estimating the supramaximal metabolic demand and anaerobic energy production are conceptually similar (di Prampero, 1986; Andersson et al., 2017). If compared, these methods would therefore likely yield identical O_2_ deficit values. However, no previous study appears to have employed the EC method for estimating anaerobic energy production. A direct practical advantage of using EC rather than GE is that external power output does not need to be determined when calculating EC. For example, both GE and/or EC have been observed to be independent of power output and/or speed in trained athletes at submaximal exercise intensities >60% of $\dot{\text{V}}$O_2max_ during cycle ergometry and diagonal-stride (DS) cross-country roller-skiing (Ransom et al., 2008; de Koning et al., 2012; Andersson et al., 2017). It is therefore likely that both GE and EC determined from a single, submaximal exercise bout could be used to estimate the O_2_ deficit during supramaximal DS roller-skiing, similar to the GE method used in cycling (Serresse et al., 1988; Noordhof et al., 2011).

Despite clear computational differences between the GE and MAOD methods, only one direct comparison has examined the O_2_ deficits obtained by these two methods (Noordhof et al., 2011). In their study, Noordhof et al. (2011) compared three different discontinuous MAOD models using protocols conducted over 2 days with the GE method using a one-off, 6-min submaximal cycling bout. No significant differences were observed between the O_2_ deficits estimated from the different models, but the most reliable estimates of O_2_ deficit were obtained from the 4 × 4-min MAOD method and the GE method. In addition, the GE method required only one submaximal exercise bout and was, therefore, considerably more time-efficient than the MAOD method. Given the sparsity of comparisons currently available in the research literature, the aim of the current study was to compare estimates of the accumulated O_2_ deficits obtained from a continuous, DS cross-country roller-skiing protocol completed on a single test day using a range of different models: the 4 × 4-min MAOD method with (method 1) and without (method 2) the inclusion of a Y-intercept, and the GE (method 3) and EC (method 4) methods using one intensity vs. four intensities. It was hypothesized that the GE/EC methods using one submaximal stage vs. four stages would result in similar values of the estimated O_2_ deficit.

## Methods

### Participants

Eleven male and 10 female junior cross-country skiers (age: 17.5 ± 1.4 years, height: 173.9 ± 8.7 cm, body mass: 68.2 ± 9.7 kg) competing at a national and/or international level were recruited from two specialist ski schools. Testing was performed off-season, starting 2 weeks after the last ski competition, while the athletes were still completing ~8–9 h of endurance training and two gym-based training sessions per week. The skiers were instructed to abstain from alcohol for at least 24 h before testing and from caffeine on the day of the trial before testing. All athletes had experience of treadmill roller-skiing and the test protocols as part of their seasonal training and performance monitoring. The study was pre-approved by the Regional Ethical Review Board of Umeå University, Umeå, Sweden and all participants were fully informed of the nature of the study before providing written consent. Additional parental consent was obtained for those under 18 years.

### Study overview

Participants were tested twice on a treadmill under laboratory conditions on a single test day, employing the DS cross-country skiing sub-technique during all testing. The first test served as a pre-test to the uphill (7°) 600-m time trial (TT) and was used to obtain baseline and submaximal $\dot{\text{V}}$O_2_ data, as well as to determine $\dot{\text{V}}$O_2max_. The 600-m TT was completed ~2.5 h after the pre-test. Participants arrived at the laboratory on the morning of testing in a fed and rested state, having completed only light training on the previous day. They were familiarized to the TT twice in non-experimental training sessions before the test day.

### Equipment and measurements

All tests were performed on a motor-driven treadmill designed for roller-skiing (Rodby Innovation AB, Vänge, Sweden). Height and body mass of the participants, as well as the mass of the roller-skis, were measured before testing (Seca 764, Hamburg, Germany). Pro-Ski C2 classical roller skis (Sterners, Dala-Järna, Sweden) with a rolling-resistance coefficient of 0.021 ± 0.001 (mean ± SD) were used. The rolling resistance of the skis was determined as described previously by Ainegren et al. (2008). Before testing, all roller-skis were pre-warmed for at least 60 min in a heating box to avoid changes in resistance of the wheels and bearings due to a warming-up effect. For safety reasons participants wore a safety harness around their waist that was suspended from the ceiling and connected to an emergency brake. Self-pacing during the TT was possible with lasers that automatically increased (0.50 km·h^−1^·s^−1^) or decreased (0.40 km·h^−1^·s^−1^) the speed if the athlete moved to the front or rear of the belt, respectively, maintaining a constant speed otherwise (Swarén et al., 2013). Respiratory variables were measured using an AMIS 2001 model C ergospirometry system (Innovision A/S, Odense, Denmark). Gas analysers were calibrated with a mixture of 16.0% O_2_ and 4.5% CO_2_ (Air Liquide, Kungsängen, Sweden) and calibration of the flowmeter was performed at low, medium and high flow rates with a 3-L air syringe (Hans Rudolph, Kansas City, MO, USA). Ambient conditions were monitored with an external apparatus (Vaisala PTU 200, Vaisala Oy, Helsinki, Finland) and the laboratory temperature was 18.0 ± 0.3°C during all testing. Heart rate (HR) was monitored continuously (RS800CX, Polar Electro Oy, Kempele, Finland) during all testing and blood lactate concentration was measured from a fingertip sample collected 3 min after the $\dot{\text{V}}$O_2_ max test (Biosen 5140, EKF diagnostic GmbH, Magdeburg, Germany).

### Testing procedures

Following a 4-min warm-up, participants performed 4 × 4-min continuous, submaximal stages at a fixed incline of 7° with $\dot{\text{V}}$O_2_ measured continuously. The warm-up and first stage were completed at 5.2–7.0 km·h^−1^ and the treadmill speed was increased thereafter by either 0.8 or 1.0 km·h^−1^ per stage, up to final speeds of 7.6–10.0 km·h^−1^. A fixed value for baseline $\dot{\text{V}}$O_2_ of 5.1 mL·kg^−1^·min^−1^ was used as the Y-intercept in the linear speed-$\dot{\text{V}}$O_2_ relationship, based on previous observations made by Medbø et al. (1988) using a group of participants with a similar $\dot{\text{V}}$O_2max_. Following the submaximal test and a 1-min break, participants completed the exhaustive $\dot{\text{V}}$O_2max_ portion of the test. The $\dot{\text{V}}$O_2max_ test started at 10, 11, or 12 km·h^−1^ and 3° or 4° and involved increases in treadmill incline every minute up to a maximum of 9°, after which speed was increased by 0.4 km·h^−1^ every minute until participants were unable to continue (i.e., could no longer match the speed of the treadmill). The highest 30-s moving average was used to calculate $\dot{\text{V}}$O_2max_, maximal ventilation rate, respiratory exchange ratio and maximal HR. The selected speeds during the submaximal test and $\dot{\text{V}}$O_2max_ test were based on sex, ability of the participant and previous test results. After a 2.5-h break participants completed the 600-m TT, which commenced with a 15-min warm-up followed by a 1-min break, then the self-paced TT (McGawley and Holmberg, 2014). At the end of the maximal tests (i.e., the $\dot{\text{V}}$O_2max_ and TT tests), a rating of perceived exertion (RPE) was recorded.

### Calculations

#### Submaximal roller-skiing

External power output (PO) was calculated as the sum of the power exerted to elevate the body mass and skiing equipment (m_tot_) against gravity and to overcome rolling resistance:

(1) $$PO\;\left[W\right]=\;m_{tot}\times g\;(\sin\left(\alpha \right)\;+u_{R}\times \cos\left(\alpha \right))\times v$$

where *g* is gravitational acceleration, *v* is the treadmill speed [m/s], μ_*R*_ is the rolling resistance coefficient and α is the treadmill incline (Andersson et al., 2017). The metabolic rate (MR) was determined from $\dot{\text{V}}$O_2_ (L·min^−1^), respiratory exchange ratio (RER: $\dot{\text{V}}$CO_2_/$\dot{\text{V}}$O_2_) and gross energy expenditure (E_gross_), where E_gross_ was calculated according to the equation introduced by Weir (1949):

(2) $$E_{gross}\;\left[Kcal\cdot \min^{-1}\right]=\left(1.1\times RER+3.9\right)\times \dot{V}O_{2\;}[L\cdot \min^{-1}]$$

To convert E_gross_ to MR the equation was modified as follows:

(3) $$MR\;[J\cdot s^{-1}]=(E_{gross}\times 4184)÷60$$

The MR was based on the $\dot{\text{V}}$O_2_ during the final 30 s of each stage in the submaximal tests. The relative energy cost (EC_rel_) of submaximal roller-skiing was calculated as:

(4) $$EC_{rel}\left[J\cdot kg^{-1}\cdot m^{-1}\right]=MR÷m_{tot}÷v$$

The GE and net efficiency (NE) during the final 30 s of each stage in the submaximal tests were calculated using the following equations:

(5) GE [%]=(PO÷MR)×100

(6) $$NE\;[\%]=(PO÷\left(MR-MR_{BL}\right))\times 100$$

where MR_BL_ is the baseline MR calculated from a fixed baseline $\dot{\text{V}}$O_2_ of 5.1 mL·kg^−1^·min^−1^ (Medbø et al., 1988) and an RER value of 0.85, reflecting normal respiratory values at rest (Haff and Dumke, 2012). The delta efficiency (%) was calculated by dividing the delta increase in PO by the delta increase in MR based on the linear regression between MR and PO over the four intensities (i.e., the reciprocal value of the slope of the regression equation).

#### Estimating the O_2_ deficit

For the MAOD method, the linear relationship between treadmill speed at 7° and $\dot{\text{V}}$O_2_ during the final 30 s of each of the four 4-min submaximal stages was derived for each individual with the fixed baseline $\dot{\text{V}}$O_2_ as a Y-intercept (i.e., a fixed value for $\dot{\text{V}}$O_2_ at zero speed) included in (4+Y) or excluded from (4-Y) the model. The Y-intercept in the 4+Y model was based on all five data points in the regression (i.e., not forced). The two regression equations were used to estimate the $\dot{\text{V}}$O_2_ demand (in mL·kg^−1^·min^−1^) at the individual average speed attained during the TT. In addition, a similar method was used where the linear relationship between $\dot{\text{V}}$O_2_ and speed was modified to a linear relationship between MR (see Equations 2, 3) and speed. The required metabolic rate (MR_req_) during the supramaximal TT could then be estimated by using the regression equation and converted to a $\dot{\text{V}}$O_2_ demand by using the following equation:

(7) $$\begin{array}{l} \dot{V}O_{2}\;demand\left[mL\cdot kg^{-1}\cdot \overset{-1}{\min}\right]=(MR_{req\;}\left[J\cdot s^{-1}\right]\times TT_{time}[s])\\ \;÷\;20.92\;[J\cdot mL^{-1}\;VO_{2}]\;÷m_{tot}[kg]÷TT_{time}[\min] \end{array}$$

where TT_time_ is the time to complete the 600-m TT. Assuming a 100% carbohydrate utilization during supramaximal exercise (i.e., a RER value of 1.00), using the energetic equivalent for 1 L of consumed O_2_ according to Weir (1949). For the GE method, the MR_req_ during the supramaximal TT was calculated by dividing the average PO (as described in Equtaion 1) by the pre-determined GE according to the criteria described previously by Andersson et al. (2017), where the average GE value from the 4 x 4-min submaximal stages was used if the individual GE-speed linear regression was found to be independent of speed (i.e., *r*^2^ < 0.50). If velocity dependency was observed (*r*^2^ ≥ 0.50) the equation of the linear regression was used for prediction of the supramaximal GE. In addition, the individual relationship between speed and GE was only considered as being speed dependent if the relative effect of speed on GE was >1.1% (i.e., a 1.1% change of the GE quotient per 1 km·h^−1^ increase in speed) together with an *r*^2^ ≥ 0.50. An analogous method to the GE method is the use of submaximal EC (see Equation 4) to estimate the supramaximal MR_req_ using the following equation (modified from di Prampero (2003)) and the same criteria for speed dependency/independency as described for GE:

(8) $$MR_{req\;}[J\cdot s^{-1}]=(EC\;[J\cdot m^{-1}]\times TT_{dist}\;[m])÷TT_{time}[s]$$

where TT_dist_ is the 600-m TT distance. For both the GE and EC methods the MR_req_ during the TT was converted to a $\dot{\text{V}}$O_2_ demand (in mL·kg^−1^·min^−1^) according to Equation (7). For all methods described, the total accumulated O_2_ deficit (in mL) was given by subtracting the accumulated VO_2_ from the accumulated VO_2_ demand during the TT.

### Statistics

The Statistical Package for the Social Sciences (SPSS 21, IBM Corp., Armonk, NY, USA) was used to carry out statistical analyses and the level of significance was set at α ≤ 0.05. Data were checked for normality by visual inspection of Q-Q plots and histograms together with the Shapiro-Wilks analysis and are presented as mean ± standard deviation (SD), except in the case of HR and RPE, where data are presented as median and interquartile range (IQR). One-way repeated measures ANOVA tests with a Bonferroni α correction were used to analyze the $\dot{\text{V}}$O_2_ demands and anaerobic capacities determined from the two MAOD methods (i.e., 4+Y and 4-Y) and the GE/EC method. The assumption of sphericity was tested using Mauchly's test. Partial eta squared ($\eta_{\text{P}}^{2}$) effect size (ES) values were also reported. The bias ± 95% limits of agreement were evaluated for the four methods (i.e., 4+Y, 4-Y, GE and EC) by using Bland-Altman calculations (Bland and Altman, 1986). The bias was tested with a one-sample *t*-test using a reference value of zero. Relationships between variables were assessed using linear regression and Pearson's correlation analyses. The individual delta efficiencies, correlation coefficients, standard error of estimates (SEE), Y-intercepts and mean slopes based on the two different linear regressions (i.e., 4+Y and 4-Y) were compared with a paired *t*-test. In addition, the relative and absolute typical error for the comparisons were computed by taking the SD for the pair-wise mean differences (as a percentage and absolute values) divided by the square root of two.

## Results

The skiers completed the incremental test to exhaustion in 6.5 ± 1.2 min and reached a $\dot{\text{V}}$O_2max_ of 61.2 ± 7.1 mL·kg^−1^·min^−1^ (males: 67.2 ± 2.6 mL·kg^−1^·min^−1^; females: 54.7 ± 3.5 mL·kg^−1^·min^−1^), a maximal ventilation rate of 157.3 ± 33.4 L·min^−1^, respiratory exchange ratio of 1.17 ± 0.05 and a maximal HR of 202 (IQR = 197–206) beats·min^−1^. The RPE on completing the $\dot{\text{V}}$O_2max_ test was 19 (IQR = 19–20) and the blood lactate concentration after 3 min was 11.0 ± 2.1 mmol·L^−1^.

**Figure 1:**
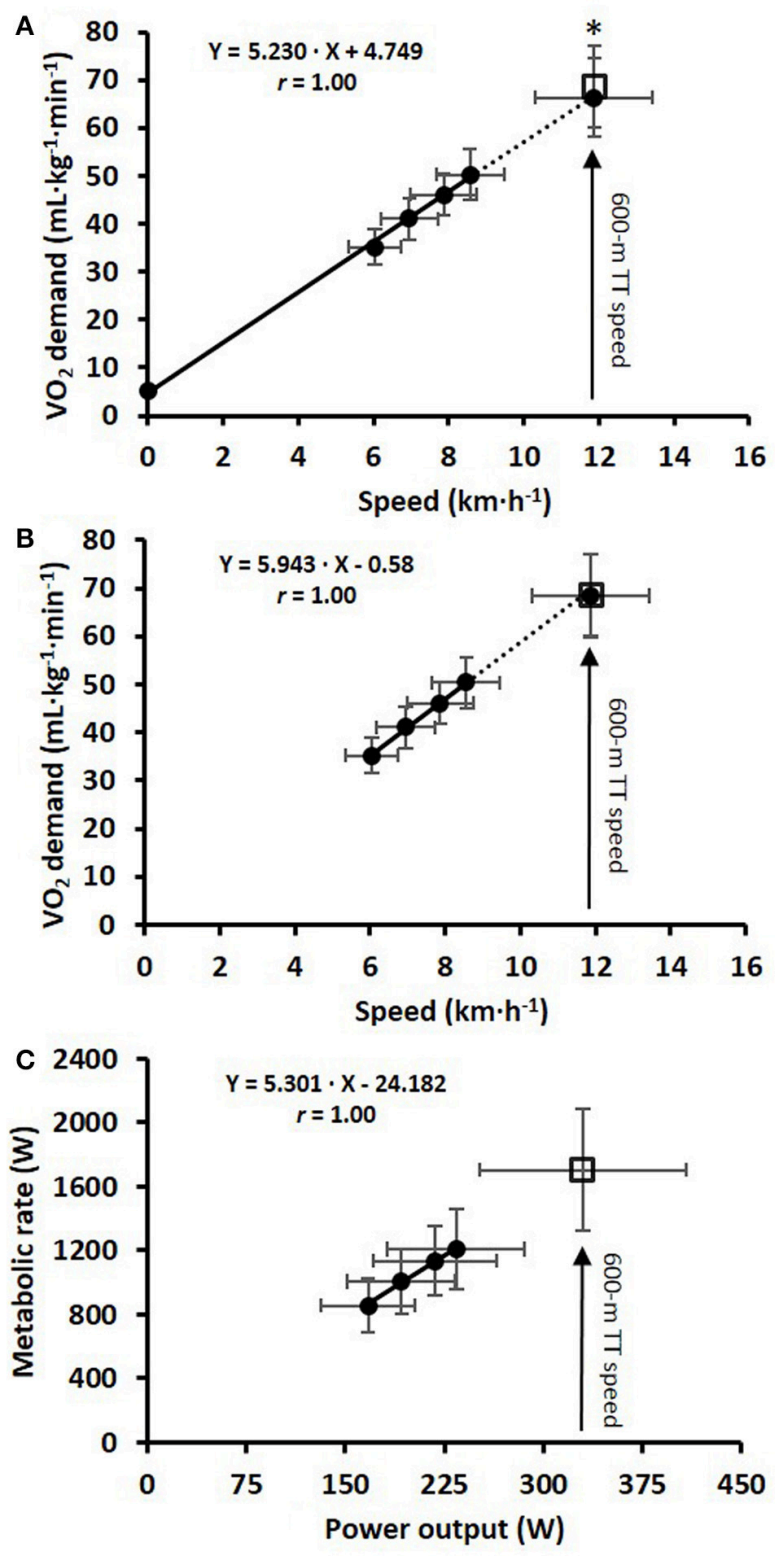
**(A)** The linear relationship between mean ± SD treadmill speed and $\dot{\text{V}}$O_2_ during 4 × 4-min of submaximal diagonal roller-skiing at 7° using the Y-intercept (4+Y), together with the estimated $\dot{\text{V}}$O_2_ demand at the average speed attained during the 600-m time-trial (TT); **(B)** the same relationship without the use of a Y-intercept (4-Y); **(C)** the linear relationship between metabolic rate and power output for the same 4 × 4-min stages of submaximal diagonal skiing. The open square represents the supramaximal $\dot{\text{V}}$O_2_ demand estimated with the gross efficiency (GE) method. ^*^Significant difference (*P* < 0.05) between the estimated supramaximal $\dot{\text{V}}$O_2_ demands using the 4+Y and GE methods.

The cardiorespiratory variables, two various concepts of efficiency (i.e., NE and GE), together with relative EC at each of the four submaximal speeds, are shown in Table 1. Neither GE nor EC was dependent on speed, both when analyzed on an individual basis and as a group mean. The delta efficiencies for the 4+Y and 4-Y regressions between MR and PO were 21.6 ± 0.9 and 19.1 ± 1.1%, respectively (*P* < 0.05). The mean ± SD of $\dot{\text{V}}$O_2_ during the four stages, together with the extrapolated $\dot{\text{V}}$O_2_ demand during the TT and the associated estimate using the GE model, are displayed in Figure 1A for the 4+Y model and Figure 1B for the 4-Y model. The relationship between MR and PO for the submaximal roller-skiing, together with the estimated supramaximal metabolic demand during the TT computed using the GE method, are presented in Figure 1C. The individual linear regressions between $\dot{\text{V}}$O_2_ (mL·kg^−1^·min^−1^) and speed (km·h^−1^) for the 4+Y and 4-Y methods demonstrated correlation coefficients of *r* = 1.00 ± 0.00 and 0.99 ± 0.01, SEE of 0.8 ± 0.4 and 0.7 ± 0.5 mL·kg^−1^·min^−1^, Y-intercepts of 4.9 ± 0.1 and 1.4 ± 2.8 mL·kg^−1^·min^−1^, and mean slopes of 5.2 ± 0.2 and 5.6 ± 0.3 mL·kg^−1^·min^−1^ per km·h^−1^, respectively (*P* < 0.05 for 4+Y vs. 4-Y for SEE, Y-intercept and slope).

**Table 1:** Mean ± SD of speeds, heart rates, cardiorespiratory variables, efficiencies and relative energy costs associated with the four submaximal stages (SUB_1–4_) of diagonal roller-skiing at 7°.

	**SUB_1_**	**SUB_2_**	**SUB_3_**	**SUB_4_**
Speed (km·h^−1^)	6.0 ± 0.7	7.0 ± 0.8	7.9 ± 0.9	8.6 ± 0.9
Heart rate (% of maximum)	80 ± 3	85 ± 3	89 ± 3	93 ± 2
$\dot{\text{V}}$O_2_ (mL·kg^−1^·min^−1^)	36.4 ± 3.8	42.9 ± 4.4	47.5 ± 4.4	50.5 ± 5.3
$\dot{\text{V}}$O_2_ (% of $\dot{\text{V}}$O_2max_)	60 ± 5	70 ± 4	78 ± 5	82 ± 5
Respiratory exchange ratio	0.92 ± 0.03	0.93 ± 0.03	0.96 ± 0.02	0.98 ± 0.02
Ventilation rate (L·min^−1^)	67.8 ± 11.7	81.6 ± 15.1	96.0 ± 17.0	105.3 ± 18.0
Gross efficiency (%)	19.5 ± 0.9	19.1 ± 0.7	19.1 ± 0.7	19.3 ± 0.8
Net efficiency (%)	22.9 ± 1.2	21.8 ± 1.0^*^	21.5 ± 0.8^*^	21.5 ± 1.0^*^
Relative energy cost (J·kg^−1^·m^−1^)	7.2 ± 0.3	7.3 ± 0.3	7.3 ± 0.3	7.3 ± 0.3

*$\dot{\text{V}}$O_2_, oxygen uptake; $\dot{\text{V}}$O_2max_, maximal oxygen uptake. Statistical comparisons were performed for efficiencies and energy cost*.

* Significantly different from the first stage (P < 0.05)

The participants completed the TT in 185 ± 24 s, with an average speed of 11.9 ± 1.6 km·h^−1^ and an average PO of 330 ± 78 W (males: 164 ± 9 s, 13.2 ± 0.7 km·h^−1^, 5.1 ± 0.3 W/kg, 390 ± 44 W; females: 208 ± 10 s, 10.4 ± 0.5 km·h^−1^, 4.0 ± 0.2 W/kg, 264 ± 47 W). The maximal HR during the TT was 196 (IQR = 192–200) beats·min^−1^ with an RPE value reported immediately after the TT of 19 (IQR = 18-19). Based on the estimated $\dot{\text{V}}$O_2_ demands during the TT, the TT speed/power output corresponded to exercise intensities of 112 ± 5, 116 ± 5, and 116 ± 5% of $\dot{\text{V}}$O_2max_ for the 4+Y, 4-Y, and GE methods, respectively. Applying the EC method resulted in identical estimated O_2_ deficit values as for the GE method. The $\dot{\text{V}}$O_2_ demands and O_2_ deficits calculated using the three methods (4+Y, 4-Y, and GE/EC) are shown in Table 2.

**Table 2:** Mean ± SD of oxygen uptake ($\dot{\text{V}}$O_2_) demands and oxygen (O_2_) deficits associated with the 600-m diagonal roller-skiing time trial at 7° using four different methods of calculation.

	**Method of calculation**				
	**4+Y**	**4-Y**	**GE/EC**	***F*-value**	**ES**
$\dot{\text{V}}$O_2_ demand (mL·kg^−1^·min^−1^)	66.3 ± 8.2^a^^b^	68.4 ± 8.7	68.7 ± 8.5	*F*_(2, 40)_ = 30.6^*^	0.61
O_2_ deficit (mL·kg^−1^)	56 ± 10^a^^b^	62 ± 10	63 ± 10	*F*_(2, 40)_ = 35.9^*^	0.64

*4+Y and 4-Y, the 4 × 4-min maximal accumulated O_2_ deficit methods with the fixed baseline $\dot{\text{V}}$O_2_ as a Y-intercept either included (4+Y) or excluded (4-Y); GE/EC, the gross efficiency/energy cost method based on the average of four submaximal stages*.

*F-values, P-values, and ES (partial eta squared effect size, $\eta_{\text{P}}^{2}$) were obtained by a one-way ANOVA*.

* *Main effect between methods (P < 0.05)*.

a *Statistically significantly different from 4-Y (P < 0.05)*.

b *Statistically significantly different from GE/EC (P < 0.05)*.

**Figure 2:**
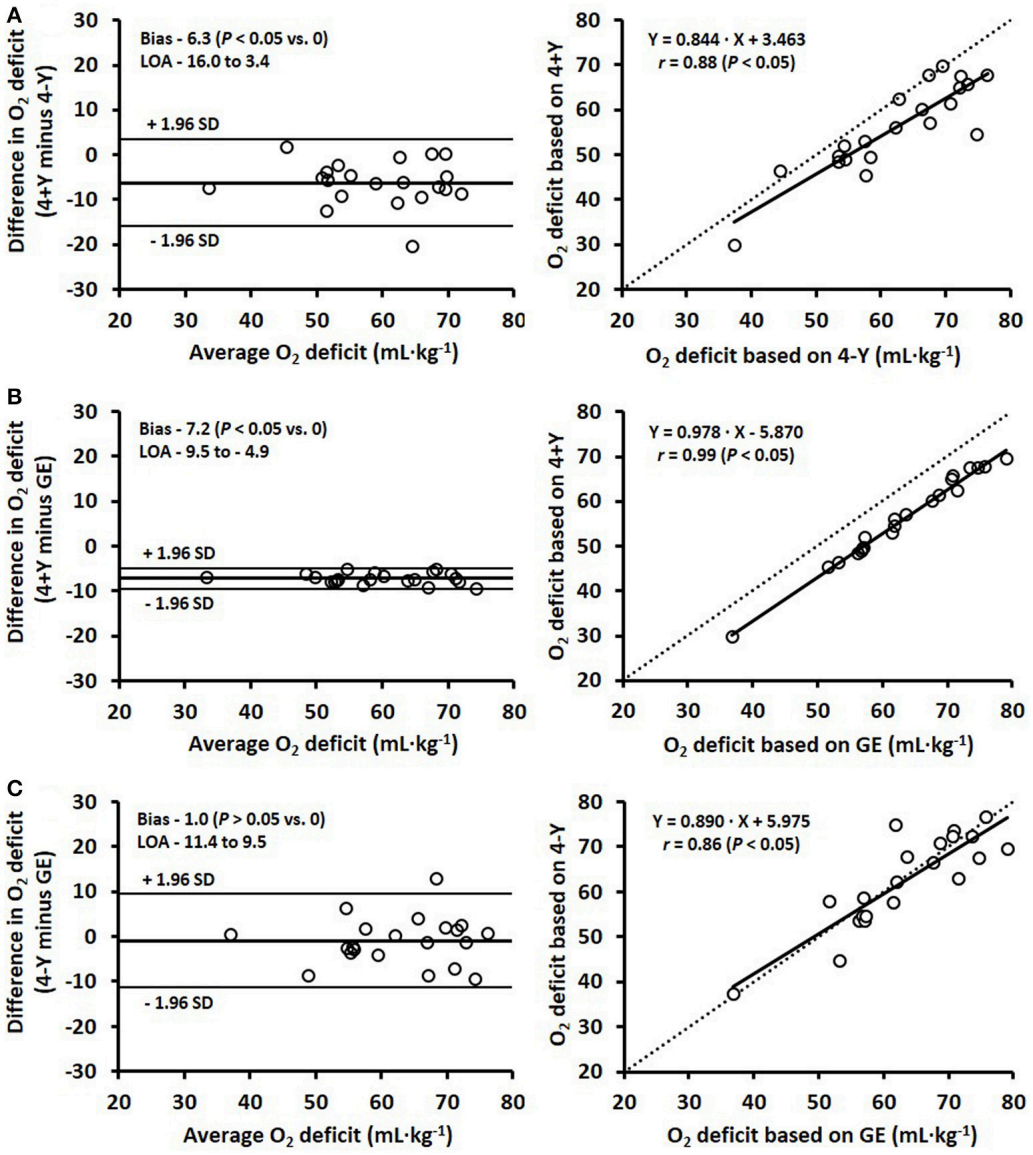
Bland-Altman plots (left) and corresponding scatter plots (right) for the estimated O_2_ deficits comparing three different methods: **(A)** 4+Y vs. 4-Y, **(B)** 4+Y vs. gross efficiency (GE [the average of four stages]), and **(C)** 4-Y vs. GE, where 4+Y and 4-Y represent the 4 × 4-min maximal accumulated O_2_ deficit methods with the fixed baseline $\dot{\text{V}}$O_2_ as a Y-intercept either included (4+Y) or excluded (4-Y). Bland-Altman plots represent the mean difference in O_2_ deficit (i.e., systematic bias) ± 95% (1.96 SD) limits of agreement (LOA) between the methods. Lines of identity are shown on the scatter plots by dashed lines.

The 95% limits of agreement and scatter plots for the comparisons between the three models are presented in Figure 2. The mean difference (i.e., systematic bias) between the O_2_ deficit estimated with the 4+Y vs. 4-Y method was −6.3 ± 4.9 mL·kg^−1^, with the 4+Y vs. GE/EC method was −7.2 ± 1.2 mL·kg^−1^ and with the 4-Y vs. GE/EC method was −1.0 ± 5.3 mL·kg^−1^, with respective typical errors of 5.3% (3.5 mL·kg^−1^), 1.9% (0.8 mL·kg^−1^), and 6.0% (3.8 mL·kg^−1^).

**Figure 3:**
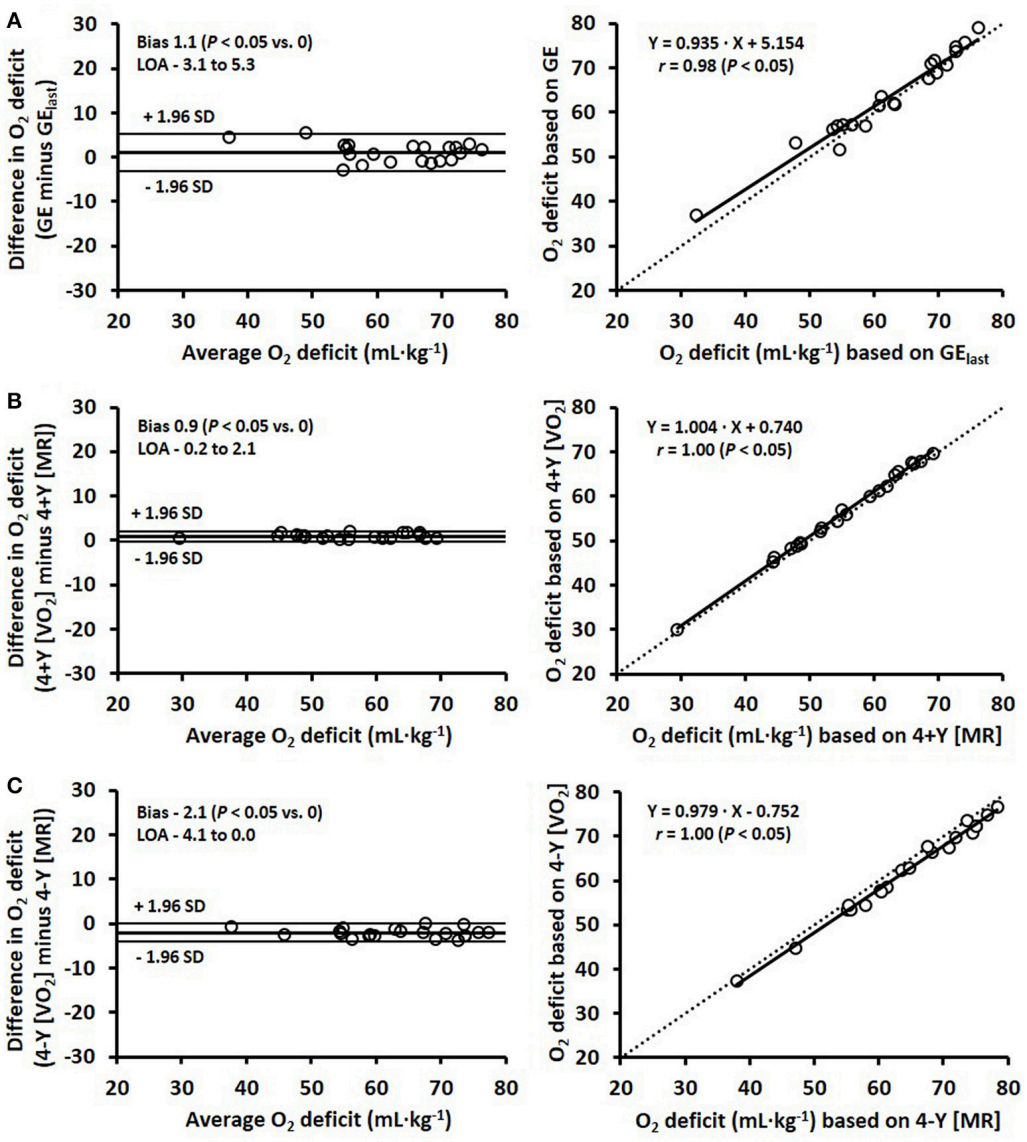
Bland-Altman plots (left) and corresponding scatter plots (right) for the estimated O_2_ deficits comparing three different methods: **(A)** gross efficiency (GE) as the average of the four submaximal stages vs. GE calculated from the last stage (GE_last_), **(B)** 4+Y [$\dot{\text{V}}$O_2_] vs. 4+Y [MR], and **(C)** 4-Y [$\dot{\text{V}}$O_2_] vs. 4-Y [MR], where 4+Y and 4-Y represent the 4 × 4-min maximal accumulated O_2_ deficit methods with the fixed baseline $\dot{\text{V}}$O_2_ as a Y-intercept either included (4+Y) or excluded (4-Y) and based on a linear regression between $\dot{\text{V}}$O_2_ and speed [$\dot{\text{V}}$O_2_], and metabolic rate and speed [MR]. Bland-Altman plots represent the mean difference in O_2_ deficit (i.e., systematic bias) ± 95% (1.96 SD) limits of agreement (LOA) between the methods. Lines of identity are shown on the scatter plots by dashed lines.

The mean difference between the O_2_ deficit estimated with GE/EC based on the average of four submaximal stages compared with the last submaximal stage was 1.1 ± 2.1 mL·kg^−1^ (Figure 3A), with a respective typical error of 3.2% (1.5 mL·kg^−1^). The comparisons between the two MAOD methods (4+Y and 4-Y) using linear extrapolation of the $\dot{\text{V}}$O_2_-speed relationship (4+Y and 4-Y [$\dot{\text{V}}$O_2_]) and the MR-speed relationship (4+Y and 4-Y [MR]) are presented in Figures 3B,C. The mean difference between the O_2_ deficit estimated with the 4+Y [$\dot{\text{V}}$O_2_] and 4+Y [MR] method was 0.9 ± 0.6 mL·kg^−1^, and between the 4-Y [$\dot{\text{V}}$O_2_] and 4-Y [MR] method was −2.1 ± 1.0 mL·kg^−1^, with respective typical errors of 0.7% (0.4 mL·kg^−1^) and 1.1% (0.7 mL·kg^−1^).

## Discussion

The main findings of the current study were that the estimated supramaximal $\dot{\text{V}}$O_2_ demand during a 600-m DS roller-skiing TT was 3% lower when a fixed value for baseline $\dot{\text{V}}$O_2_ was included in the MAOD method (i.e., 4+Y, method 1) as compared to no inclusion of baseline $\dot{\text{V}}$O_2_ (i.e., 4-Y, method 2) and the GE/EC methods (methods 3 and 4). The higher Y-intercept in the 4+Y vs. 4-Y method resulted in an 8% lower slope of the regression line. Although the estimated values of O_2_ deficit between the four methods were highly correlated (*r* = 0.86–0.99), the limits of agreements ranged from 5 to 21 mL·kg^−1^ and typical errors ranged from 1.9 to 6.0%, indicating that the different methods should not be used interchangeably. Moreover, since GE/EC was independent of speed, the O_2_ deficits estimated with the GE/EC methods using one submaximal stage vs. four stages were highly related (*r* = 0.98) and highly similar (bias of 1 mL·kg^−1^), as hypothesized.

The MAOD method has been deemed valid for estimating the O_2_ deficit during isolated knee-extension exercise (Bangsbo et al., 1990). Nevertheless, there is currently no gold standard for estimating the O_2_ deficit during whole-body exercise and several different MAOD approaches have been used (Green and Dawson, 1993; Noordhof et al., 2010). One main discrepancy when using the MAOD method appears to be how the linear relationship between submaximal $\dot{\text{V}}$O_2_ and speed is constructed. Inconsistencies in the literature relate to the duration, intensity and number of stages included in the modeling, as well as whether a continuous or discontinuous exercise protocol should be used (Green and Dawson, 1993, 1996; Noordhof et al., 2010). In the current study, a continuous 4 × 4-min protocol was employed incorporating relatively high exercise intensities (60–82% of $\dot{\text{V}}$O_2max_). This was based on previous findings showing no differences in the estimated $\dot{\text{V}}$O_2_ demand when using continuous vs. discontinuous protocols (Green and Dawson, 1996), or whether more than four stages are included in the linear regression (Bickham et al., 2002). Relatively high submaximal intensities were used in the present study, in an attempt to minimize the error of estimating the $\dot{\text{V}}$O_2_ demand by extrapolation, which is likely related to the magnitude of the difference in intensities between the measured and predicted $\dot{\text{V}}$O_2_ demand at the supramaximal intensity (Bangsbo, 1998). One drawback of using high submaximal intensities is the potential for an increased anaerobic energy yield to affect the submaximal linear relationship, thereby leading to an underestimation of the $\dot{\text{V}}$O_2_ demand (Green and Dawson, 1993; Noordhof et al., 2010). However, the participants in the current study were well-trained endurance athletes and the RER values during the submaximal intensities were < 1.00. In addition, the DS skiing sub-technique cannot be performed effectively at low speeds (Andersson et al., 2017).

A forced Y-intercept using either $\dot{\text{V}}$O_2_ measured at baseline or an arbitrary value has previously been applied in the MAOD method for increasing the precision of the estimated $\dot{\text{V}}$O_2_ demand (Medbø et al., 1988; Russell et al., 2000, 2002; Bickham et al., 2002). However, in the present study where DS roller-skiing was employed as the exercise mode, the inclusion of a fixed value for baseline $\dot{\text{V}}$O_2_ (i.e., method 1) resulted in a lower $\dot{\text{V}}$O_2_ demand compared with the other methods (2–4). The modeled Y-intercept resulting from the 4-Y method in the current study was 1.4 mL/kg/min, which is considerably lower than the modeled Y-intercept of 4.9 mL/kg/min for the 4+Y method involving a fixed value for baseline $\dot{\text{V}}$O_2_ of 5.1 mL/kg/min, based on previous suggestions by Medbø et al. (1988). This supports previous findings of an exponential $\dot{\text{V}}$O_2_ response from baseline (i.e., at rest) up to high submaximal speeds (Barstow and Mole, 1991; Green and Dawson, 1995; Bangsbo, 1996b, 1998). Moreover, the degree of non-linearity between $\dot{\text{V}}$O_2_ and speed varies between different forms of locomotion and between participants of different fitness levels, due to a non-linear variation in O_2_ cost that can be partly explained by the $\dot{\text{V}}$O_2_ slow component (Green and Dawson, 1995; Billat et al., 1998; Pringle et al., 2002; Noordhof et al., 2010). Since the magnitude of the $\dot{\text{V}}$O_2_ slow component is related to both duration and intensity of exercise, its potential influence on the estimated $\dot{\text{V}}$O_2_ demand cannot be excluded in the current study. However, the $\dot{\text{V}}$O_2_ slow component is generally low in endurance athletes (Jones et al., 2011) and has been shown to be markedly reduced after a period of intensified endurance training in untrained subjects (Womack et al., 1995). Therefore, the combination of using well-trained endurance athletes as participants and employing a relatively short submaximal test (4 × 4-min) would probably limit the magnitude of any developing slow component and its influence on the linear equations (4-Y and 4+Y methods) used to estimate the supramaximal $\dot{\text{V}}$O_2_ demand.

In cross-country skiing, anaerobic capacity has been estimated using both the MAOD (Losnegard et al., 2012; McGawley and Holmberg, 2014; Sandbakk et al., 2016) and GE methods (Andersson et al., 2016, 2017). However, in agreement with previous findings by Noordhof et al. (2011), the results presented in the current study revealed a relatively high level of disagreement between the MAOD and GE/EC methods, suggesting that they should not be used interchangeably. The disagreement between the analyzed methods (4+Y, 4-Y, and GE/EC) can be associated with computational differences and may to some extent also be related to the different concepts of efficiency. The 4-Y method is relatively similar to delta efficiency, whereas the 4+Y method is more similar to the concept of net efficiency, as baseline $\dot{\text{V}}$O_2_ is taken into account (Noordhof et al., 2011). The 8% lower slope of the regression line between $\dot{\text{V}}$O_2_ and speed when including a baseline $\dot{\text{V}}$O_2_ (i.e., 4+Y) resulted in a delta efficiency that was 2.5 percentage points higher than for the 4-Y method, which explains the differences in the estimated $\dot{\text{V}}$O_2_ demands between the two methods. Although the typical error for the differences in O_2_ deficits estimated with the 4+Y vs. GE/EC was low (1.9%), and results were highly correlated (*r* = 0.99), a high systematic bias was observed between the methods with significantly lower O_2_ deficit values (7.2 mL·kg^−1^) for the 4+Y method. In contrast to the present findings, Noordhof et al. (2011) observed no significant differences in the estimated O_2_ deficits between the 4+Y, 4-Y, and GE methods. This inconsistency in findings might be caused by factors that influence the degree of linearity between $\dot{\text{V}}$O_2_ and speed from baseline (i.e., $\dot{\text{V}}$O_2_ at zero speed), which relate to different exercise modalities (cycling vs. DS roller-skiing) and the wider range of submaximal stages (30–90% of $\dot{\text{V}}$O_2max_) employed in the study by Noordhof et al. (2011). Diverse results between the two studies may also be related to the fact that Noordhof et al. (2011) used GE calculated from one submaximal stage and not, as in the current study, an average GE based on all submaximal stages as submaximal intensities < ~60% of $\dot{\text{V}}$O_2max_ were considered too low for reflecting GE at a supramaximal effort.

It is well known that endurance-trained athletes are able to metabolize a higher relative amount of fat than untrained individuals during submaximal exercise (Kiens et al., 1993) and that fat requires ~7% more oxygen than carbohydrate at a similar MR (Weir, 1949). However, this factor of unsystematic error is not considered when constructing a linear relationship between submaximal $\dot{\text{V}}$O_2_ and speed with the traditional MAOD method and the alternative method presented in the current study, where MR is plotted against speed, is potentially more accurate for estimating a supramaximal $\dot{\text{V}}$O_2_ demand (or MR_req_). This is supported by previous findings of Green and Dawson (1995), which showed that up to 46% of the difference in submaximal $\dot{\text{V}}$O_2_ at a given PO between well-trained cyclists and untrained participants could be related to differences in substrate utilization, which also influences the $\dot{\text{V}}$O_2_-power regressions. However, in the group of athletes recruited in the current study, differences in substrate utilization exerted only a slight impact on the estimated O_2_ deficit (Figures 3B,C), but this potential error might be amplified in heterogeneous groups with more diverse fitness levels.

The GE method is commonly used for estimating anaerobic work and/or power during supramaximal exercise (Noordhof et al., 2011). However, GE can only be calculated if the external work can be defined, which constitutes a direct problem for activities like walking and running (van Ingen Schenau and Cavanagh, 1990). Therefore, the EC method presented in the current study offers an alternative method of estimating the O_2_ deficit during running exercise, as well as an advantage during roller-skiing on fixed treadmill gradients in that external work does not need to be determined. In the current study, GE and EC during submaximal DS roller-skiing were found to be independent of speed, both when analyzed individually and on a group level, which is similar to previous observations by Andersson et al. (2017). Moreover, the agreement between the O_2_ deficits estimated with GE based on the average from four submaximal stages and the GE based on the last submaximal stage was relatively high (bias of 1.1 ± 2.1 mL·kg^−1^, *r* = 0.98). Therefore, the findings of the current study suggest that only one submaximal stage may be needed (probably at a relatively high submaximal intensity, i.e., 70–80% of $\dot{\text{V}}$O_2max_) when using the GE and/or EC methods to estimate O_2_ deficit during supramaximal exercise, provided GE/EC is found to be independent of speed. The constant GE/EC observed in the current study for DS roller-skiing at relatively high submaximal exercise intensities (i.e., 60–82% of $\dot{\text{V}}$O_2max_), is similar to previous observations of well-trained athletes during cycle ergometry (Ransom et al., 2008) and treadmill running (di Prampero, 1986). These findings are of potential practical relevance, since estimating anaerobic energy production using GE or EC calculated from a single submaximal stage is more time-efficient than the classical MAOD procedure introduced by Medbø et al. (1988). Therefore, the different methods compared in the current study require further investigation in other exercise modes and/or sub-techniques in cross-country skiing.

At present, there is no gold standard method for estimating anaerobic capacity during whole-body exercise (Noordhof et al., 2010, 2013). A problem with both the MAOD and GE/EC methods is the potential for a significant anaerobic contribution during the submaximal exercise, which would result in an underestimated $\dot{\text{V}}$O_2_ demand and hence O_2_ deficit. Therefore, future studies should, in combination with $\dot{\text{V}}$O_2_ measurements, include continuous blood lactate sampling (Björklund et al., 2011) during the submaximal exercise bouts to obtain a more accurate estimation of the metabolic/$\dot{\text{V}}$O_2_ demand through the use of energetic equivalents for changes in blood lactate concentration (di Prampero and Ferretti, 1999). A fundamental problem with the MAOD approach lies in the construction of the linear $\dot{\text{V}}$O_2_-speed relationship during submaximal exercise. Although a continuous submaximal protocol, as used in the current study, is more time-efficient than discontinuous protocols conducted over several days (Medbø et al., 1988; Noordhof et al., 2011), a continuous protocol may be problematic due to a gradually increasing $\dot{\text{V}}$O_2_ slow component, with the possibility for overestimating the supramaximal $\dot{\text{V}}$O_2_ demand (Noordhof et al., 2010). Since endurance-trained athletes have a considerably reduced $\dot{\text{V}}$O_2_ slow component (Jones et al., 2011), a continuous submaximal protocol used within the MAOD method would probably be more problematic in a group of untrained individuals. Other problems may relate to changes in movement economy and/or GE during the supramaximal exercise (Noordhof et al., 2013), which have recently been observed during supramaximal cycle ergometry time trials (Noordhof et al., 2015). For instance, if a decreasing GE during the supramaximal work is evident but not confirmed in the method, the O_2_ deficit will be underestimated, which constitutes a potential limitation of the present study as well as studies using the conventional GE and MAOD methods (Noordhof et al., 2013, 2015). One direct methodological advantage of using the GE/EC method presented here, rather than the MAOD approach, is that estimated changes in GE/EC during supramaximal efforts can be incorporated into the model for estimating the $\dot{\text{V}}$O_2_ demand and hence O_2_ deficit, as proposed by Noordhof et al. (2015).

In the current study, external power output was based solely on power causing propulsion similar to previous studies (Sandbakk et al., 2011, 2016; Andersson et al., 2016, 2017). The approach employed in the current study differs from the mechanical power calculation used by Pellegrini et al. (2014) and Kehler et al. (2014) in that internal mechanical power was not considered. Since the calculation of external power output, in the current study, was based on a balance between the propulsive forces generated and all opposing forces, notably those associated with gravity and rolling resistance, the values obtained are not exact. Although a direct measure of the instantaneous propulsive power during roller-skiing on a dual-belt force-instrumented treadmill would probably be more accurate (Kehler et al., 2014), such a set-up was not possible in the present study. The main problems with an indirect estimation of external power output during DS roller-skiing are in part related to the unloading of the roller-skis during the poling phase, and that a part of the movement cycle is not subjected to rolling resistance on two wheels (Pellegrini et al., 2014). Therefore, it is likely that our indirect approach has overestimated the power against rolling resistance. However, since only 14.6% of the total external power output (or work) was due to power against rolling resistance, this possible error was probably relatively low. This error would probably have a relatively similar effect on the external power output calculation across all of the studied exercise intensities and hence only influence the O_2_ deficit calculation minimally when using the GE method.

In summary, the primary aim of the current study was to compare four different methods of estimating anaerobic energy production during supramaximal uphill DS roller-skiing. Secondary intentions were to introduce new methodological concepts and to compare these with currently existing methods. Although the MAOD method has been suggested as having the potential to indirectly quantify anaerobic energy production (Bangsbo et al., 1990; Saltin, 1990), no standardized method currently exists. One disadvantage of the traditional 10 × 10-min discontinuous submaximal protocol for constructing the $\dot{\text{V}}$O_2_-speed relationship suggested by Medbø et al. (1988) is that it is very time-consuming. The various methods presented in the current study are more beneficial from a practical perspective and the O_2_ deficit estimates were highly correlated. However, the level of bias and relatively high typical errors suggest that the different methods should not be used interchangeably, except the GE and EC methods, which produced identical results and may therefore be used interchangeably. Since GE/EC was found to be independent of speed during DS roller-skiing on a treadmill in highly trained athletes, the current findings indicate that only one submaximal stage may be needed for estimating anaerobic energy production. This could be included in a warm-up before a maximal test, for example, so may from both methodological and practical perspectives be preferable to the traditional MAOD method for estimating anaerobic capacity. However, this novel proposition requires further investigation and the one-stage method requires validation across a variety of exercise modes before it can be recommended as standard practice.

## Author contributions

EA and KM designed the study and KM collected data. EA performed the data and statistical analyses. EA and KM interpreted the results, wrote the draft and revised the manuscript. EA and KM approve the final version to be published and agree to be accountable for all aspects of the work.

### Conflict of interest statement

The authors declare that the research was conducted in the absence of any commercial or financial relationships that could be construed as a potential conflict of interest.
